# Activating waitlists: Identifying barriers and facilitators to pain self-management while waiting

**DOI:** 10.1177/20494637241311456

**Published:** 2025-01-06

**Authors:** Lydia V Tidmarsh, Richard Harrison, Harriet Wilkinson, Megan Harrington, Katherine A Finlay

**Affiliations:** 1 School of Psychology and Clinical Language Sciences, 6816 University of Reading, Reading, UK; 2 Centre for Integrative Neuroscience and Neurodynamics, 6816 University of Reading, Reading, UK; 3 156748 Royal Berkshire Hospital, Reading, UK

**Keywords:** Behaviour change, health-locus of control, pain management, prehabilitation, qualitative, reflexive thematic analysis

## Abstract

**Objectives:**

Waitlists for pain management services are often extensive, risking psychological and physical decline and patient non-engagement in treatment once accessed. Currently, for outpatient pain management, no standardised waiting list interventions exist, resulting in passive waiting. To arrest prospective wait-related decline(s), this study aimed to identify the barriers and facilitators to pain self-management while waiting, forming the foundation for a waitlist intervention development.

**Design:**

An inductive qualitative approach was utilised to explore the barriers and drivers of pain self-management while waiting for chronic pain management.

**Method:**

Semi-structured interviews, underpinned by the Theoretical Domains Framework and COM-B model, were conducted with people waiting for pain management services (*N* = 38). Interviews were audio-recorded, transcribed verbatim, and analysed via reflexive thematic analysis.

**Results:**

The analysis demonstrated four thematised barriers and one facilitator: (1) Shunted Around the System *(barrier)*; (2) The Information Gap *(barrier)*; (3) Resisting Adaptation (*barrier*); (4) Losing Hope (*barrier);* and (5) Help Yourself or Lose Yourself *(facilitator)*.

**Conclusion:**

This study demonstrates the severe emotional and motivational impact of waiting, increasing treatment disengagement. The waitlist represents a prime opportunity for prehabilitation to protect wellbeing and optimise self-management engagement. Infrastructural and interpersonal barriers of poor communication and healthcare professional pain invalidation must be addressed to improve emotional wellbeing and motivation to engage with planned treatment. Enhancing self-efficacy, pain acceptance, self-compassion, and internal HLOC are fundamental to increasing pain self-management. These can all be met within a prehabilitation framework. This study is foundational for the development of psychological prehabilitation in outpatient chronic pain management.

## Introduction

Globally, healthcare services are under significant economic and resource strains, resulting in extensive waitlists.^1^ In chronic pain management, recommended waiting times are a maximum of two-months for non-urgent, and one-month for urgent cases.^2^ However, currently, global pain management timelines more typically range between eight months and two years^3,4^; risking severe detriment for people living with chronic pain (PLwCP) waiting for treatment. Significantly elevated levels of stress, pain, functional impairment, anxiety and depression are associated with long treatment delays.^5–9^ These detrimental effects are rapid; psychological decline can occur within five weeks of waiting,^10^ therefore average pain management wait-times drastically exceed this critical time-window. Treatment attrition consequently becomes problematic, as waiting for four months or longer reduces the likelihood of not attending treatment by 25%,^11^ diminishing treatment cost-effectiveness.^12^ Dissatisfaction with care may ensue, with PLwCP feeling abandoned and lost within the system.^3,13^ As a result, PLwCP may become disengaged and demotivated, risking non-attendance for pain management services. As persistent pain requires active participation by the individual to manage their condition,^14^ when physiological/psychological decline occurs and treatment motivation drops, self-management success is likely to be reduced. It is therefore clear that there is an urgent need to prospectively arrest this decline.

To address this growing concern, the Faculty of Pain Medicine^15^ recommends earlier application of pain management principles. Pain management aims to empower PLwCP to be able to self-manage their pain.^16^ Targeting behaviour change to encourage engagement in pain self-management during the waitlist, as opposed to passive waiting, would activate the waiting period, transforming it from one of decline to preparation. Psychological factors including negative expectations, low self-efficacy, pain catastrophizing and external health-locus of control (HLOC) interact, influencing attrition, treatment engagement and outcomes during waiting (*see review*).^17^ Importantly, these are all central constructs within Pain Management Programme (PMP) content.^16^ Negative expectations regarding pending treatment are associated with lower self-efficacy, and greater pain catastrophizing and pain intensity following psychological intervention.^18^ Incongruent patient expectations regarding treatment outcomes are also associated with attrition.^19^ Pain catastrophizing, defined as a set of maladaptive cognitive biases towards pain,^20^ is typically elevated while waiting for treatment.^10^ This is especially concerning as higher pain catastrophizing at pre-treatment is predictive of low engagement in PMPs^21^ and attrition.^22^ By contrast, self-efficacy and HLOC are directly associated,^23^ where patients with more internal HLOC display higher pain self-efficacy, and greater competence and engagement in pain self-management strategies.^24^ Critically, these factors are known to be amenable to change through targeted intervention.^13,25–29^

For effective behaviour change intervention, firstly, a detailed assessment of the behavioural targets (what needs to change) is required in the specified context and population.^30^ Behaviour change interventions are more successful when they are theoretically derived.^31^ The key theoretical stances for behavioural intervention development combine the COM-B model^30^ and the Theoretical Domains Framework (TDF).^32^ These two comprehensive frameworks are increasingly applied in parallel, identifying at a granular level, barriers and drivers of behavioural change, with an action-oriented approach.^33–35^ Utilising these theoretical un derpinnings, establishing pain self-management as the target behaviour, and chronic pain patients on a waitlist for pain management as the target group, this research aims to explore the research question: When awaiting treatment, how do chronic pain patients characterise the facilitators and barriers to pain self-management?

## Method

### Design

A semi-structured qualitative interview design was used to identify perceived facilitators and barriers to pain self-management during the waiting list time period, from the patient perspective.

### Participants

PLwCP on the waitlist for pain management at Royal Berkshire Hospital NHS Trust were invited to take part in the study. A total of 38 participants completed the interviews, aged between 20 and 87 years (M = 51, SD = 15.23). The inclusion criteria for participation were participants aged 18 years or above, on the formal clinical waiting list of the Pain Management Unit (PMU) and a diagnosis of chronic pain. Participant demographics are presented in Table 1.

**Table 1.** Participant demographics.

Total	*N* = 38
Age (years)	M = 51.3, SD 15.2
	Range 20 – 81
Sex	Male = 10 (26%)
	Female = 28 (74%)
Ethnicity	
White British	32 (84%)
Asian - Other Asian Background	3 (8%)
Black or Black British	2 (5%)
Asian or Asian British - Indian	1 (3%)
Employment status	
Employed	15 (39.5%)
Unable to work due to pain	10 (26.3%)
Unemployed	2 (5.3%)
Retired	6 (15.8%)
Unknown	5 (13.1%)
Pain condition	
Fibromyalgia	11 (28.9%)
Osteoarthritis	8 (21.1%)
Nerve pain	4 (10.5%)
CRPS	4 (10.5%)
Musculoskeletal pain	3 (7.9%)
Rheumatoid arthritis	2 (5.3%)
Pelvic pain	2 (5.3%)
Sciatica	2 (5.3%)
Annular tear lumbar spine	2 (5.3%)
Mean duration of chronic pain (years)	10.1
Range duration of chronic pain (years)	0.5 – 30
Mean time spent waiting before interview (days)	104
Range time spent waiting before interview (days)	7 – 444

### Materials

A 17-item semi-structured interview schedule (see Table 2) was created exploring facilitators and barriers to pain self-management while waiting. Intervention development research recommends both an action-oriented and a behavioural theoretical perspective.^35–37^ Therefore, the interview schedule was informed by the TDF^32^ and COM-B model,^30^ providing a behavioural science theoretical grounding to the interview questions, with the aim to facilitate future targeted behaviour change.

**Table 2.** Interview schedule.

COM-B construct	COM-B micro-construct	Theoretical domain	Interview questions
	Introductory question		1. What does the phrase ‘self-management of pain’ mean to you?
Capability	Psychological	Knowledge	2. What knowledge and information do you personally draw on when you’re trying to manage your pain by yourself?
		Behavioural regulation	3. If you were going to improve your ability to manage your pain yourself, what do you think you would need to change?
		Memory/attention/decision process	4. You are currently at [an early/later] stage on the waiting list for the Pain Management Programme. Thinking of your pain *now*, how do you decide whether or not to use specific techniques or approaches to manage your pain?
	Physical	Skills	5. What practical skills do you think you need to improve your ability to manage your pain?
Opportunity	Physical	Environmental context and resources	6. What environmental factors or resources currently affect your ability to manage your pain?
			7. How do the pain management services you’ve received so far, either from the NHS or privately, impact your ability to self-manage your pain?
	Social	Social influences	8. How do the people around you influence the ways in which you manage your pain yourself?
Motivation	Reflective	Beliefs about capability	9. To what extent do you see managing your pain yourself as part of *your* role?
			10. How confident do you feel in your ability to manage your pain yourself at the moment?
		Optimism	11. To what extent are you optimistic about your ability to manage your pain yourself in the future?
		Beliefs about consequences	12. How much benefit do you think you would gain from being able to improve your ability to manage your pain?
		Intentions	13. What motivates you to try to manage your pain yourself?
		Goals	14. What, if any, are your current goals for improving your ability to manage your pain yourself?
	Automatic	Reinforcement	15. What, if any, habits or patterns do you spot in your approaches to managing your pain yourself?
		Emotion	16. How does it affect you *emotionally* when you’re trying to apply your *current* techniques/approaches to manage your pain yourself?
	Closing question		17. When you’re on the waiting list for a pain management programme, what additional support would you want from the pain management team to help you through that waiting period?

### Procedure

People who were on the waiting list for the PMU were invited to participate in the study via email. Information sheets were provided, and participants were informed their involvement in the study would have no influence on their clinical care/care pathway, and that they had the right to withdraw at any time without reason. Following provision of written informed consent, one-to-one semi-structured interviews were conducted either face-to-face (*N* = 5), or online via Microsoft Teams (*N* = 33), depending on participant preference. The chosen data analysis strategy was reflexive thematic analysis (RTA), therefore when determining the sample size, data saturation was not employed (following gold standard RTA methodology recommendations).^38^ Rather, data quality was reviewed during the process of data collection via monthly review meetings with the research team to determine the information power, encompassing the adequacy of the data regarding its richness and complexity in answering the research question.^39^ Data collection ceased when it was determined information power was achieved. Transcripts were anonymised, assigned pseudonyms and obscuring all identifiable features. Signposts to support services were provided in the information sheet, and participants describing previous/current suicidal ideation (*N* = 4) were flagged to the clinical team for follow-up. The duration of the interviews ranged between 31 and 103 min (M = 55.7, SD = 14.8). The study was approved by the University of Reading ethics committee (UREC: 22/06) and the United Kingdom Health Research Authority (*HRA/22/NW/0059, IRAS 302397*).

### Data analysis

Interviews were audio-recorded online and transcribed verbatim using Otter.ai by the first author (LVT). Following accuracy checking of typographical correction, transcripts were analysed according to all six phases of Braun & Clarke^40^ reflexive thematic analysis (RTA): (1) familiarisation with the data; (2) initial coding; (3) generating initial themes; (4) developing and reviewing themes; (5) refining, finalising, and naming themes; and (6) writing up. The distinct phases are recursive; the reflective nature of the analysis meant that phases were repeated during the process of interpretation and meaning seeking.^41^ Reflexive thematic analysis was selected due to the ability to identify rich patterns of meaning across large datasets encapsulating participants’ lived experience, while also acknowledging the role of the researcher as an active agent in meaning searching and shaping.^42,43^ RTA is inherently interpretative, valuing the interaction of the researcher within analysis, thus there is no endpoint where codes could cease to form.^41^ NVivo 12 was utilised to code and organise the data. Phase 1 involved the first author familiarising with the data by repeated listening of the transcripts. Next, in phase 2, initial coding of the transcripts was conducted representing barriers and facilitators to pain self-management. Initial codes were then clustered depending on their demonstrated semantic or conceptual similarity for phase 3. Thereafter, initial, and subsequent higher-order themes were generated (phase 4) after shaping various iterations to ensure each theme was clearly focused and boundaried (phase 5). For credibility and trustworthiness, coding was undertaken by two researchers^44^ (LVT and KAF). The shared meaning of codes, sense checking, theme narratives and the overlaps and divergencies of identified themes were discussed by the whole research team during theme refinement.^44,45^ Where disagreements occurred, consensus was reached through consultation with the transcripts and in-depth discussion with the research team. Thematic headings were discussed in depth and considered to increase resonance of the findings.^46^

A critical realist epistemological position was adopted, underpinning the analysis. Such epistemology acknowledges that a reality exists, while recognising the role of individual perception, as understanding is always contextual.^43^ As RTA emphasises researcher subjectivity as a resource,^41^ the research team continually reflected on the potential impact of their individual experiences when analysing and reviewing themes. All authors are Psychologists who have experience working in hospital settings and managing chronic health conditions. Regular discussions throughout data analysis between the research team employed a critical reflection strategy, with RH acting as a critical friend throughout the data analysis process.^36,38^ The analysis was conducted using an inductive approach, whereby there was no direct attempt to fit the data into existing theory. However, as the interview schedule was informed by the TDF and COM-B model, this likely will have indirectly influenced coding of the data.^47^ To minimise this occurring, field notes were kept during interviews and reflective practice was maintained by weekly reviews with the research team.

## Results

**Figure 1.**
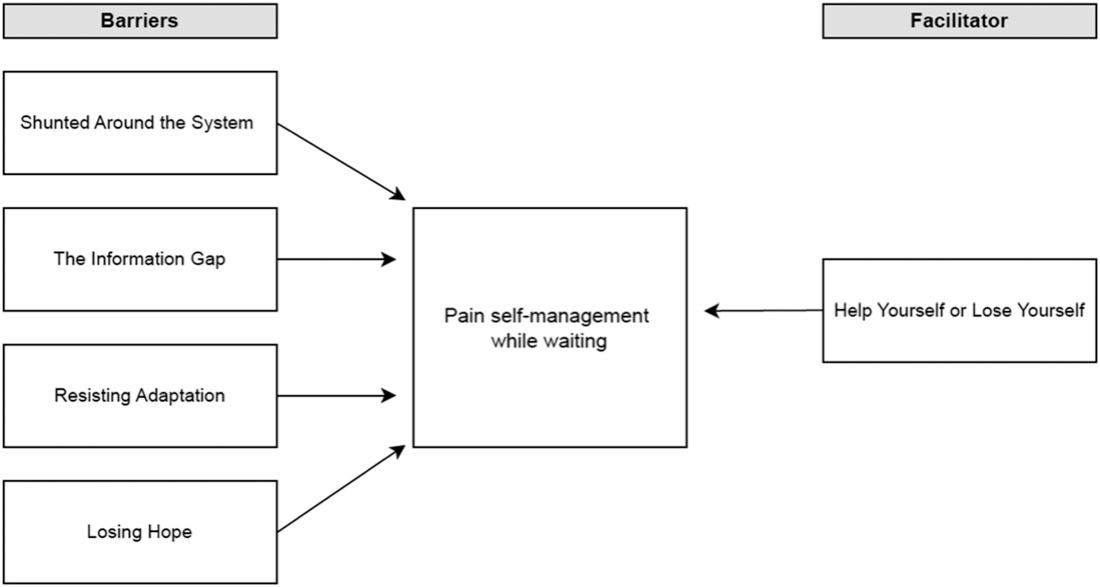
Thematic map.

Results from inductive reflexive thematic analysis demonstrated that four thematised barriers and one thematised facilitator were represented in the data: (1) Shunted Around the System *(barrier)*; (2) The Information Gap *(barrier)*; (3) Resisting Adaptation *(barrier);* (4) Losing Hope (*barrier);* and (5) Help Yourself or Lose Yourself *(facilitator*; see Figure 1 for a thematic map).

### Shunted around the system (barrier to pain self-management)

There was a prevailing theme of feeling shunted between clinical departments, and the feeling of being moved on to become someone else’s problem. The complexity of chronic pain means that, often, before reaching pain specialists, PLwCP may encounter many other specialists for investigations into the potential causes of their pain, prior to diagnosis and identification of the most appropriate treatment pathway. For PLwCP, this can feel like pain is too difficult to treat, thus (hospital) departments do not want to deal with them: *‘“Oh, you’ve got depression*. *Oh, [I] can’t deal with that one*.” *You feel shunned and pain’s a bit the same’ (Harry)*. People feel dismissed and that they are battling against the system to receive appropriate support:

‘What I get upset [about] the most is the consultants; they all want to avoid me. Orthopaedics will say this is a neurology problem. Neurology will say this is so and so’s problem. Because of a number of symptoms, they don’t know which department I quite fit just from the political point of view. And I go to see one consultant after waiting six months for an appointment, and then they’ll say, “Oh, you shouldn’t have come to see me, you should have gone somewhere else because you suffer from say arthritis and actually, we’re dealing with carpal tunnel”’ (Yoon).

Interactions with some clinicians also leave people feeling disbelieved and that they need to prove that their condition is bad enough to be heard and receive treatment: *‘I just don’t know where to go with it because I feel like I’m not being believed… I’ve talked to the nurses, to doctors, to the point where I’m in tears*. *I’m saying please, somebody’s got to help me’ (Debbie)*. Inference that chronic pain is a result of psychological causes contributes to the perception of being disbelieved: *‘If someone suggests, it’s in your head, it makes you think that I’m imagining it’ (Louise)*.

Contributing to this feeling of dismissal, participants described how they have received little or no communication regarding how long they will be waiting, how the referral process works or what they are even waiting for. This created a sense of abandonment, with participants feeling lost within the system, reducing trust or optimism in the service: *‘I’m waiting a year down the line with no one making any contact*. *So, I had given up on it’ (Ade)*. To improve a sense of care and acknowledgement, participants desired communication regarding anticipated wait-times, referral processes, what pain management entails and how it can help PLwCP. This would help PLwCP to make an informed decision regarding whether the treatment pathway is something they want to engage in:

‘What is a pain management programme exactly? How long is it going to take? What does it involve? What they could do for me. Because for all I know, I could be waiting for nothing’ (Yoon).

### The information gap (barrier to pain self-management)

During what felt like uncertain and endless waiting, participants expressed a two-fold ‘information gap’ impacting their ability to self-manage their pain; (1) regarding techniques relevant to their daily struggles; and (2) how chronic pain manifests and persists to support pain validation.

Participants described an insufficient information provision regarding pain self-management techniques they could implement in their daily life, relevant to their personal difficulties. Engaging with lower impact physical activity was a specific area on which participants desired lifestyle information. Without being informed of the correct type and execution of physical exercise, this instilled a barrier to attempting such due to a fear of physical deterioration: *‘Someone said about Aqua aerobics, but is it going to damage the lower part of my spine even more? Is it gonna damage me?’ (Karen)*. Gaining information regarding pain management strategies, outside of medication, was important to participants in order to insulate them from further decline:

‘I told my GP I’m not depressed, I’m in pain. I’m desperate, not depressed. But I don’t want antidepressants, I want treatment. So, more education and skills on how to self-regulate, meditate or hypnosis, stretching, yoga, physiotherapy – things you can do outside of opioids’ (Irina).

Participants described how this information is withheld until treatment. However, having this information of self-management strategies to try while waiting would support them to self-manage their pain:

‘I was told there are techniques which they felt would help me [with sleep and pain]. Waiting, I haven’t able to get that information. If they [HCPs] did have other suggestions, it would have been helpful to try them while I was on the waiting list’ (Margaret).

Educating PLwCP on the mechanisms underlying the maintenance of chronic pain was also important for individual understanding of what chronic pain is and why it persists, facilitating self-management. Improving the way in which such information is communicated is also essential to help reduce the feelings of invalidation from healthcare professionals (HCPs):

‘When I first visited the pain clinic 15 years ago, the overwhelming sense was it was all in my head, and I walked away feeling like I wasn't believed. 15 years on, the sentence of “it's all in your head,” I agree with, but there's more depth of understanding of how pain manifests. Because the learning of pain in the last decade has changed massively’ (Amelia).

### Resisting adaptation (barrier to pain self-management)

Without strategies of how to self-manage their pain effectively, PLwCP experienced an internal conflict between their physical ability and attempts to retain their sense of self. This was largely represented in their ability to conduct daily tasks or valued activities. Reflecting on their physical ability prior to pain and self-expectations of what they ‘should’ be able to do, led to resisting adaptation for pain management. This resulted in continued activity despite consequences of worsened pain and becoming debilitated:

‘I wouldn’t let [the pain] stop me from doing what I wanted to do. And this is what people are saying, that is your problem, you will not allow yourself to stop. But why should I?’ (Janet).

In attempts to retain their identity, participants expressed how pride can be an influential factor in maintaining behavioural patterns, and at times, denying their condition. Asking for help can exacerbate the sense of vulnerability already felt due to their declining physical condition. Resistance to help-seeking impaired asking for support, sometimes refusing help or adaptation for pain management:

*‘Your pride and dignity and everything else sort of goes out the window a bit*. *You’re losing a little bit more*. *I think it’s pride because you don't want to admit that you actually are in that situation [of needing help]*. *I'm a proud woman, and I don't like to burden people with my issues’ (Annabelle)*. Such resistance results in ‘boom and bust’, further exacerbating pain and ability to self-manage: *‘I don't want to acknowledge it*. *I put my head in the sand and keep going until I can't move’ (Amelia)*.

### Losing hope (barrier to pain self-management)

Persistent high pain intensity, unsuccessful strategies, and poor emotional wellbeing reduced hope in participants’ ability to manage their pain. Low confidence, in turn, reduced motivation to continue even attempting self-management strategies:

‘When you’re trying loads of things, and you’re in bits, it’s very depressing. Because you’re just like, what am I doing wrong? I’m really trying, and this isn’t working. And I don’t know what to do next. It feels very hopeless… You’re kind of just like, what’s the point?’ (Sophia).

The emotional turmoil of unmanaged pain during extensive waiting and feeling like they were fighting to be seen and heard was overbearing. Chronic pain often means people are unable to work, applying financial pressures which are exacerbated by long treatment delay, when unfurnished with pain self-management strategies. A cyclical downward spiral of emotional distress and physical decline ensued:

‘So, it’s that [waiting, feeling disbelieved, and financial pressures] which then heightens the acuteness of your mental state, which then affects your health, which then affects your pain, which affects your day-to-day activities. The knock-on effect is absolutely massive’ (Ade).

When the emotional distress was at its highest point, participants were demoralised and defeated: *‘There’s nothing I can personally change’ (Denise)*. A desire for a physical cause and medical cure administered by professionals was also represented, rather than their personal agency in employing psychological strategies, impairing their ability to self-manage their pain: *‘I wish somebody would give me an operation and stop it*. *Then I wouldn’t have to do these things’ (John)*.

### Help yourself or lose yourself (facilitator of pain self-management)

In attempts to avoid the detrimental consequences of unmanaged pain, participants recognised the importance of pain self-management; having to help oneself, otherwise they would lose themselves to the persistent pain. They were fighting to retain their emotional wellbeing, and ability to engage with life:

‘If I let all these pains and issues get on top of me, it’s so depressing any way, I will end up feeling suicidal. Because I’m reminded every day, all day, that I cannot do this, I cannot do that. So, I have to either be strong and try and help myself, or I will lose myself’ (Yoon).

Participants explained how coming to a place of realisation of the significance of their role in self-management was central to facilitating them to move forward:

*‘I’ve gone through the “why has it happened to me?” To now “what can we do to make it better?” I used to be very, “It’s not fair, why?” Rather than ‘Ok, this is where we are, what do I need to do today to make it better?’ (Amelia)*. This realisation also encompassed the importance of ownership and responsibility over their condition and self-management: *‘I can obviously take advice from professionals, but if I don’t implement those suggestions, then it’s not going to be helpful’ (Louise)*.

However, while participants acknowledged the importance of self-management, they require support to enable them to do so:

‘Oh, [my role] is humongous. Even if my role is 95%, I need that 5%. I know it’s down to me, it’s my body, my life and I have the biggest influence on it. I’m just hoping that 5% or whatever extra help I get, will give me that ability to do more for myself’ (Susan).

## Discussion

These findings identify salient facilitators and barriers to pain self-management while waiting for treatment. Results demonstrate four thematised barriers and one facilitator represented in the data: (1) Shunted Around the System *(barrier)*; (2) The Information Gap *(barrier)*; (3) Resisting Adaptation *(barrier);* (4) Losing Hope (*barrier);* and (5) Help Yourself or Lose Yourself *(facilitator*). Pain self-management was clearly limited by experiences of feeling dismissed, disbelieved by clinicians and shunted between clinical departments – *‘*Shunted *Around the System’*. During uncertain, prolonged waiting, participants felt ill-equipped to self-manage their pain – ‘*The Information Gap’*. Insufficient information impaired employment of personalised self-management strategies and limited understanding about how chronic pain manifests and persists. Without tools or help from the system, previous behavioural patterns were maintained to retain the sense of self – ‘*Resisting Adaptation*’. The combination of persistent pain, an inability to access support, insufficient information and resisting adaptation led to a downward spiral of pain, despair and disengagement, the barrier ‘*Losing Hope*’. To mitigate the emotional/physical risks of unmanaged pain while waiting, participants recognised the significance of their role within pain self-management, the facilitator *‘Help Yourself or Lose Yourself’*.

Feeling dismissed and shunted between departments, corroborates with recent studies^3,7^; little or no communication from clinicians/hospital administrators while waiting, regarding expectations of waiting time, the referral process, and *what* they are even waiting for, led to feelings of abandonment and reduced hope. Poor communication while waiting contributes to negative expectations of service quality and outcomes,^3^ predicting high pain intensity and low self-efficacy.^18^ Self-efficacy for managing chronic conditions can be increased via enhanced (targeted) knowledge,^28,48^ subsequently improving greater pain self-management engagement.^29^ Yet, inference that chronic pain is influenced by psychological factors also contributed to feeling invalidated and disbelieved by HCPs, inducing stress, emotional decline and worsened pain.^49–52^ The link between psychological distress, physiological stress and immune system response in exacerbating and prolonging pain, must be better communicated,^53,54^ confirming our findings that PLwCP are seeking information about chronic pain mechanisms. Our data highlights the need for information provision regarding anticipated wait-times, providing an outline of treatment content and delivery. Behavioural change must concurrently target both the infrastructural barriers of communication within the healthcare system, and interpersonal barriers of pain validation between patients and HCPs. Pain validation, including authentic communication to the patient, is critical for ensuring patients feel believed by HCPs regarding their pain. Empowering patients with more information regarding their pending treatment would better facilitate collaborative care,^55^ ultimately reducing excessive healthcare utilisation. PLwCP could elect not to wait months for treatment they are unwilling to engage with. Ultimately, information must be provided to PLwCP (including pain-science/psychoeducation and psychological strategies) *before* treatment, to enhance self-efficacy while waiting. This would target the TDF constructs ‘Knowledge’ and ‘Beliefs about capabilities’ within the COM-B domains of Psychological Capability and Reflective Motivation,^30,32^ initiating theory-driven behaviour change.

*‘Resisting Adaptation’* reflects conflicting self-expectations and a ‘boom and bust’ cycle, where PLwCP struggle to change busy pre-existing behavioural patterns (*‘boom’*), risking physical debilitation, depletion and maladaptive coping (*‘bust’*).^56^ Maladaptive coping impairs pain acceptance, reduces quality of life, and increases pain severity and disability,^57,58^ whereas pain acceptance facilitates self-management.^59^ Our findings highlight the recognised need for acceptance-based and self-compassion pre-treatment strategies to improve physical and emotional wellbeing.^60–62^ The theme ‘*Help Yourself or Lose Yourself*’, indicates participant recognition of the significance of *their* role in managing their pain, a form of internal HLOC. Higher levels of internal HLOC are associated with greater pain self-management engagement in chronic conditions^63^ and reduced pain intensity following multidisciplinary intervention.^64^ Importantly, HLOC is a construct amenable to change; increased internal HLOC is observed following CBT based self-management interventions.^25^ PLwCP depict a tightrope where extensive waiting precipitates psychological decline^5,7,10^ suicidal ideation,^65^ and treatment attrition.^11^ Yet, acceptance enables self-management, and prevents escalation and early mortality.^66,67^ This study highlights HLOC as a feasible psychological target for enhancing pain self-management. Perceived behavioural control is a central construct within both TDF and COM-B domains of ‘Beliefs about capabilities’ and ‘Reflective Motivation’.^30,32^ Importantly, these are core components of the PMP framework^16^; cognitive restructuring approaches to manage distressing/restricting thoughts and enhance psychological flexibility, facilitating acceptance. The current study suggests that by harnessing these factors earlier, PLwCP will be better psychologically prepared to engage in self-management once treatment is accessed.

## Prehabilitation and future directions

These findings dynamically inform the development of applied interventional research in early pain management. This study clearly shows the severe emotional and motivational impacts of waiting and the urgent need to intervene to arrest this decline. Current practice in surgical settings includes pre-operative psychoeducation as a cost-effective, successful pre-treatment clinical intervention.^68^ This is termed prehabilitation: interventions employed from the point of diagnosis, prior to attending for prescribed treatment.^68^ Prehabilitation in pain outpatient care is not yet uniformly employed. Prehabilitation saves on average 21% of the total cost of surgical procedures from subsequently reduced healthcare usage.^69,70^ Pre-surgical *psychological* prehabilitation includes pre-operative education, behavioural instruction, cognitive behavioural strategies, and stress management reduce post-operative pain, healthcare utilisation and negative affect.^71–74^ Our findings offer strong justification for the development of a psychological prehabilitation intervention for outpatient chronic pain. The identified themes evidence the need for therapeutic strategies including increasing self-efficacy via information provision (including referral processes, expected wait-times, pain self-management strategies and pain science education), and improving HCP pain validation, self-compassion, pain acceptance and internal HLOC. Physical skills training regarding safe type and execution of low-impact exercise and/or stretching was also identified as necessary to increase physical capability to engage in activity while awaiting treatment. Thus, while this study has primarily focused on psychological prehabilitation, there is the potential to integrate this in a multimodal approach, including physical activity and psychological strategies.^73,75^ If there is a multidisciplinary capacity, this could be implemented safely. The BCW, COM-B model^30^ and TDF^32^ can clearly be utilised to comprehensively and systematically identify intervention functions, behaviour change techniques and mode of delivery for intervention design and implementation.

## Strengths and limitations

The current study was situated within the waiting lists of one NHS trust Pain Management Unit, likely shaping these findings. Research comparing waiting experiences from various NHS trusts, and internationally, to ascertain greater understanding of similarities or differences of waiting, would be a valuable extension of the study. Waitlists are likely to vary in length across trusts, which would have implications for how prehabilitation would be formatted and delivered. Exploring waiting experiences across regions would be valuable to further develop a prehabilitation intervention that could be efficiently utilised in different settings. Data relating to available social support, such as relationship status, is typically not recorded on medical records, and was not available for the majority of the sample (32/38). Therefore, future research may explore the influence of this during the waiting time, as social support is shown to influence patient activation, health beliefs and depression in PLwCP.^76^ This study included patients with a wide range of chronic pain conditions, pain duration and time spent waiting, enabling a breadth of experience to be captured. Our sample was predominantly female, as aligned with chronic pain samples; women are disproportionally affected by chronic pain.^77,78^ While the majority of participants were employed (39.5%), 26.3% reported being unable to work due to their pain condition. Future research could explore how these factors of chronic pain duration, time spent waiting, gender and employment status may influence psychological decline, pain self-efficacy and pain catastrophizing during the waiting time, impacting capability for pain management. These characteristics may provide useful stratification methods to highlight the most vulnerable to extensive waiting, requiring prehabilitation intervention. The use of reflexive thematic analysis invokes subjectivity, a recognised strength of this methodology that facilitates deep engagement with the data, generating rich meaning of the lived experience.^38^

## Conclusion

The current study vividly depicts the severe physiological and emotional impact of waiting, increasing the risk of suicidal ideation and treatment disengagement. The waitlist is the prime opportunity for prehabilitation, protecting wellbeing and optimising engagement with pain self-management. By identifying the barriers and drivers of pain self-management, this study provides a fundamental foundation for the development of psychological prehabilitation for outpatient chronic pain. The infrastructural barriers of poor communication regarding referral processes and wait-times, and the interpersonal barriers of HCP pain invalidation must be addressed to improve emotional wellbeing and motivation to engage. The intrapersonal factors of enhancing self-efficacy, shifting to pain acceptance, self-compassion, and a more internal HLOC are central to increasing pain self-management engagement. Utilising often inert waiting time to specifically enhance these factors is intuitive and for PLwCP would increase engagement in the self-management programme waited for. Results from this study would recommend that cognitive behavioural techniques, goal-setting, values, psychological content for emotional support and informational resources on wait-times, pain science education and lifestyle pain management should be explored for integration into a new prehabilitation intervention for outpatient pain management. This coalesces with the self-management interventions utilising cognitive behavioural, educational and goal-setting strategies at the treatment, peri-treatment or rehabilitation level.^79–81^ However, this would be relocated in the new context of prehabilitation, in the pre-intervention setting. Further research is required to develop a prehabilitative framework and intervention design, grounded in behavioural science for effective behaviour change. This study provides a unique insight into the experience of waiting for pain management, and critically illustrates the primary psychological targets for outpatient pain prehabilitation.
